# Differential expression of microRNAs as predictors of glioblastoma phenotypes

**DOI:** 10.1186/1471-2105-15-21

**Published:** 2014-01-18

**Authors:** Barrie S Bradley, Joseph C Loftus, Clinton J Mielke, Valentin Dinu

**Affiliations:** 1 Department of Biomedical Informatics, Arizona State University, 13212 East Shea Boulevard, Scottsdale, AZ 85259, USA; 2 Biochemistry and Molecular Biology, Mayo Clinic Arizona, 13400 E. Shea Boulevard, Scottsdale, AZ 85259, USA; 3 The Biodesign Institute, Arizona State University, 1001 S. McAllister Ave, Tempe, AZ 85287, USA

**Keywords:** MicroRNA, miR, Glioblastoma, Cell migration, Gene expression, Target prediction, Pathway analysis

## Abstract

**Background:**

Glioblastoma is the most aggressive primary central nervous tumor and carries a very poor prognosis. Invasion precludes effective treatment and virtually assures tumor recurrence. In the current study, we applied analytical and bioinformatics approaches to identify a set of microRNAs (miRs) from several different human glioblastoma cell lines that exhibit significant differential expression between migratory (edge) and migration-restricted (core) cell populations. The hypothesis of the study is that differential expression of miRs provides an epigenetic mechanism to drive cell migration and invasion.

**Results:**

Our research data comprise gene expression values for a set of 805 human miRs collected from matched pairs of migratory and migration-restricted cell populations from seven different glioblastoma cell lines. We identified 62 down-regulated and 2 up-regulated miRs that exhibit significant differential expression in the migratory (edge) cell population compared to matched migration-restricted (core) cells. We then conducted target prediction and pathway enrichment analysis with these miRs to investigate potential associated gene and pathway targets. Several miRs in the list appear to directly target apoptosis related genes. The analysis identifies a set of genes that are predicted by 3 different algorithms, further emphasizing the potential validity of these miRs to promote glioblastoma.

**Conclusions:**

The results of this study identify a set of miRs with potential for decreased expression in invasive glioblastoma cells. The verification of these miRs and their associated targeted proteins provides new insights for further investigation into therapeutic interventions. The methodological approaches employed here could be applied to the study of other diseases to provide biomedical researchers and clinicians with increased opportunities for therapeutic interventions.

## Background

Glioblastoma (GB) is the most common primary central nervous system tumor and accounts for approximately 40% of all primary malignant brain tumors. GB is a heterogeneous group of tumors associated with a very poor clinical prognosis. The median survival for patients with newly diagnosed glioblastoma is approximately 15 months and declines to approximately 8 months for patients with recurrent glioma [1,2]. The 6-month progression free survival for glioblastoma is less than 20%. The biology of malignant glioma presents significant problems for successful clinical treatment. Chief among these hurdles is the aggressive local invasion of malignant cells from the original tumor. The heightened commitment to migration and reduced proliferation of invasive glioma cells makes complete surgical resection impossible, increases their resistance to chemotherapeutic agents, and reduces the efficacy of radiation treatment, which virtually assures tumor recurrence. Improved clinical treatment will therefore ultimately require a more thorough understanding of the molecular mechanisms that regulate the invasion of glioma cells from primary tumor sites as well as the identification and specific targeting of the critical drivers of glioma invasion.

The pathobiology of GB is characterized by temporal and spatial alterations in gene expression that produce phenotypically distinct cell populations. Necrosis, micro-vascular proliferation, and increased staining for proliferation markers histopathologically characterize the highly cellular tumor core. In contrast, invasive cells at the tumor – normal brain interface exhibit a decrease in the expression of proliferation markers and a relative increase in the expression of pro-apoptotic genes [3,4]. Gene expression profiling of laser-captured micro dissected cells from paired GB patient tumor core and invasive edge established an invasion signature of genes differentially expressed in the invasive cell population. This gene set represents potential targets to limit glioma dispersal and decreased therapeutic resistance as the invasion process strongly up-regulates survival signaling pathways [4-6].

The molecular mechanisms regulating the expression of pro-invasive or pro-proliferation signaling proteins are not completely understood. One potential mechanism of post-transcriptional regulation of gene expression is through microRNAs (miRs). miRs are a highly diverse class of small (~20-22 nucleotides), non-protein coding single stranded RNA molecules that play a central role in a broad range of normal biological processes by dynamically regulating protein expression [7]. miR activity has also been linked to various cancers where miRs can function either as potential oncogenes or as tumor suppressors [8]. A potential role for a number of miRs in GB progression has been reported in recent studies [9-11]. The majority of the studies were performed with cultured glioma cell lines or primary GB patient samples to annotate global changes in miR expression, but did not investigate miR expression in distinct glioma cell populations. In the current study, we investigated a dataset of miRs collected from a matched pair of migration-restricted glioma cell and migratory glioma cell populations to identify differentially expressed miRs associated with each cell population. We conducted pathway enrichment analysis with these miRs to investigate potential associated gene targets. Signaling effectors regulated by the identified differentially expressed miRs represents a potentially rich set of targets for therapeutic development.

## Results

The differential expression (mean edge cell expression minus mean core cell expression) was down-regulated in 193 (24%), and up-regulated in 612 (76%) of the 805 miRs in our study. Of the 193 down-regulated miRs, 62 (32%) exhibited both significant FDR corrected *p*-values and a ≥ 2x fold change, while 131 (68%) did not. Of the 610 up-regulated miRs, 2 (<1%) exhibited both significant FDR corrected *p*-values and a ≥ 2x fold change, while 610 (>99%) did not. The results indicate that there is a statistically significant relationship between expression direction (down-regulated vs. up-regulated) and differential expression *p*-value / fold change (Fisher’s Exact Test Two-sided p < 0.001).

**Figure 1:**
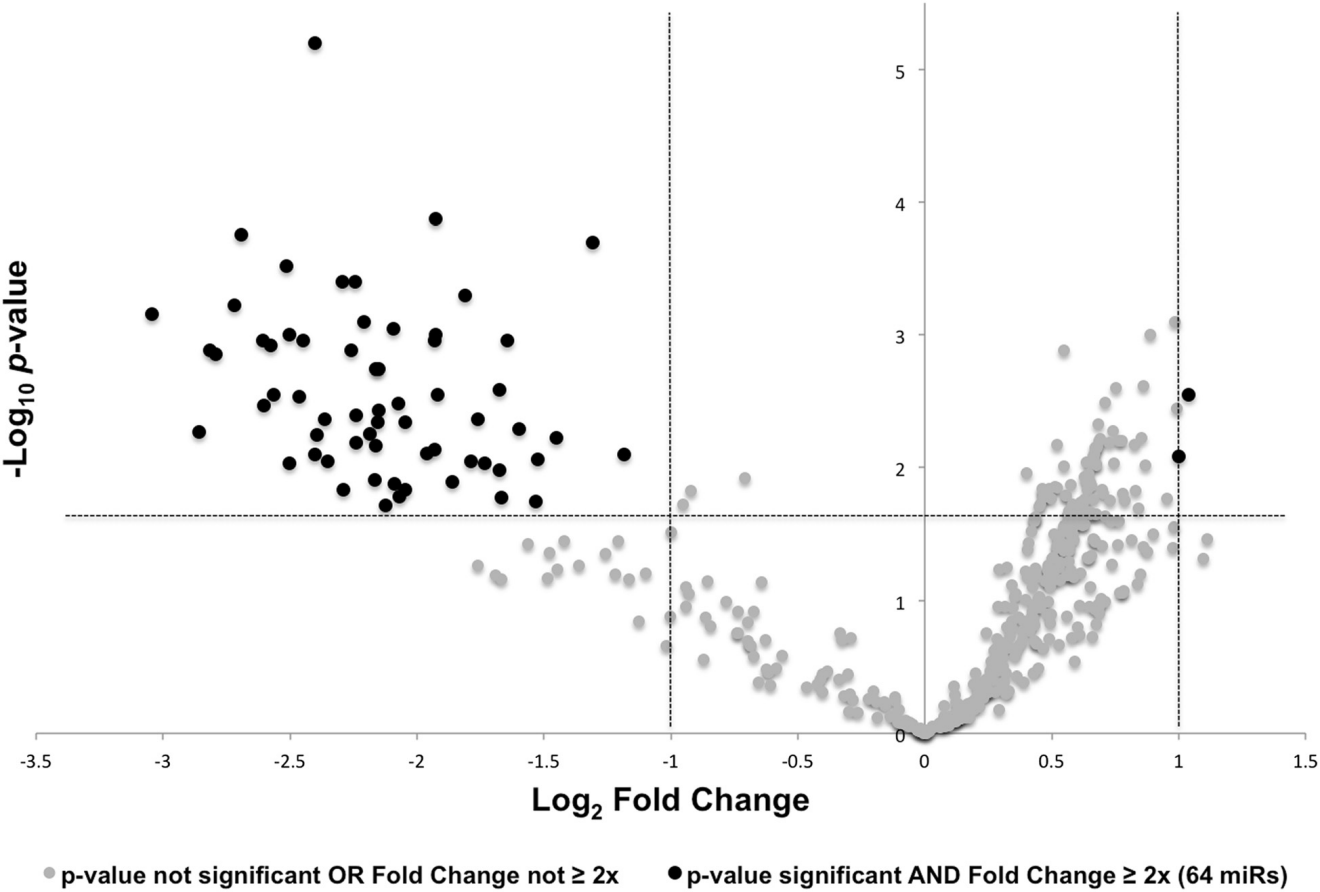
**Plot of differential expression for glioblastoma edge and core cells.** Negative Log_10_*p*-values for each of the 805 miRs were plotted on the y-axis and Log_2_ normalized fold change expression levels on the x-axis. The threshold Benjamini-Hochberg corrected -Log_10_*p*-value (1.687) is superimposed on the volcano plot for reference (horizontal dashed line) to identify miRs with significant differential expression (α = 0.05). Vertical dashed lines at -1.0 and 1.0 Log_2_ fold change represent twofold threshold values. Black dots represent the 64 miRs identified as exhibiting both a significant FDR corrected *p*-value and a ≥ twofold change in expression level.

A plot of the differential expression of these data (volcano scatter plot) illustrates graphically the distribution of miRs that are both significant (two-tailed *t*-test) and meaningful (≥ 2x fold change) (Figure 1). Those miRs located both above the FDR corrected -Log_10_*p*-value (1.69, horizontal dashed line), and a greater than 2x Log_2_ fold change (≤ -1.0 or ≥ 1.0, vertical dashed lines), are considered both significant and meaningful. Grey colored dots in the volcano plot represent those miRs that are either not significant or do not have a ≥ twofold differential expression. Black dots represent those miRs that exhibit both a significant differential expression and a ≥ 2x fold change, and thus represent those 64 miRs considered for further study (Table 1).

**Table 1:** miRs identified for study

*hsa-let-7a*	*hsa-miR-125a-5p*	*hsa-miR-197*	*hsa-miR-26a*	*hsa-miR-331-3p*
*hsa-let-7b*	*hsa-miR-125b*	*hsa-miR-19a*	*hsa-miR-27a*	*hsa-miR-365*
*hsa-let-7c*	*hsa-miR-130a*	*hsa-miR-19b*	*hsa-miR-27b*	*hsa-miR-424*
*hsa-let-7d*	*hsa-miR-140-5p*	*hsa-miR-20a*	*hsa-miR-29a*	*hsa-miR-455-3p*
*hsa-let-7e*	*hsa-miR-151-3p*	*hsa-miR-20b*	*hsa-miR-29b*	*hsa-miR-574-3p*
*hsa-let-7f*	*hsa-miR-151-5p*	*hsa-miR-21*	*hsa-miR-29c*	*hsa-miR-9*
*hsa-let-7 g*	*hsa-miR-15a*	*hsa-miR-22*	*hsa-miR-30a*	*hsa-miR-92a*
*hsa-let-7i*	*hsa-miR-15b*	*hsa-miR-221*	*hsa-miR-30b*	*hsa-miR-93*
*hsa-miR-100*	*hsa-miR-16*	*hsa-miR-222*	*hsa-miR-30c*	*hsa-miR-99a*
*hsa-miR-103*	*hsa-miR-17*	*hsa-miR-23a*	*hsa-miR-30d*	*hsa-miR-99b*
*hsa-miR-106b*	hsa-miR-17*	*hsa-miR-23b*	*hsa-miR-30e*	Up-regulated miRs
*hsa-miR-107*	*hsa-miR-181a*	*hsa-miR-24*	hsa-miR-30e*	*ebv-miR-BART15*
*hsa-miR-10b*	*hsa-miR-181b*	*hsa-miR-25*	*hsa-miR-320*	*hsa-miR-548d-5p*

A total of 62 down-regulated and 2 up-regulated miRs with significant FDR corrected *p*-value and ≥ 2x fold change.

A total of 64 miRs were identified as exhibiting both significant FDR corrected *p*-values and a ≥ 2x fold change. Of these 64 miRs, migratory edge cell expressions were down-regulated 2% of the time (mean -0.03, SD not relevant as only one such data point) and up-regulated 98% of the time (mean 1.85, SD 1.61). Migration-restricted core cell expressions were down-regulated 3% of the time (mean -0.25, SD 0.04) and up-regulated 97% of the time (mean 4.00, SD 1.91). Conversely, for the differential cell population expression, mean edge cell expression minus mean core cell expression, the differential expression was down-regulated 97% of the time (mean -2.14, SD 0.54) and up-regulated 3% of the time (mean 1.02, SD 0.18) (Table 2).

**Table 2:** Edge vs. core cell summary statistics for significant miRs

	**Frequency**		**Expression**	
**A. Edge Cells**	**n**	**%**	**Mean**	**SD**
Down-Regulated	1	2%	-0.03	NA
Up-Regulated	63	98%	1.85	1.61
Total miRs	64	100%	1.82	1.61
**B. Core Cells**				
Down-Regulated	2	3%	-0.25	0.04
Up-Regulated	62	97%	4.00	1.91
Total miRs	64	100%	3.86	1.93
**C. Differential Expression (Edge – Core)**				
Down-Regulated	62	97%	-2.14	0.54
Up-Regulated	2	3%	1.02	0.18
Total miRs	64	100%	-2.04	0.68

Significant miRs identified at α = 0.05, and utilizing a Benjamini-Hochberg false discovery rate correction.

Recent studies have demonstrated that many of the significant miRs elucidated in our study have been previously implicated in tumor migration/invasion in other cancers, including malignancies in the brain. For some of our identified miRs however, we were unable to find any prior literature that reported validated gene targets. To address this limitation, we utilized several available algorithms that predict gene targets *in silico*, such as TargetScan or PicTar. Additionally, resources are available that perform enrichment calculations on representative gene categories or biological pathways. These groups could include such categories as signal transduction, cytoskeletal organization, adhesion, apoptosis, proliferation, or transcription factors [12]. For glioma cell migration, categories such as adhesion and cytoskeletal organization would be important to study further for verification. We turned to these bioinformatics approaches to obtain a wider view of potential genes and pathways that could be targeted by these identified miRs.

**Figure 2:**
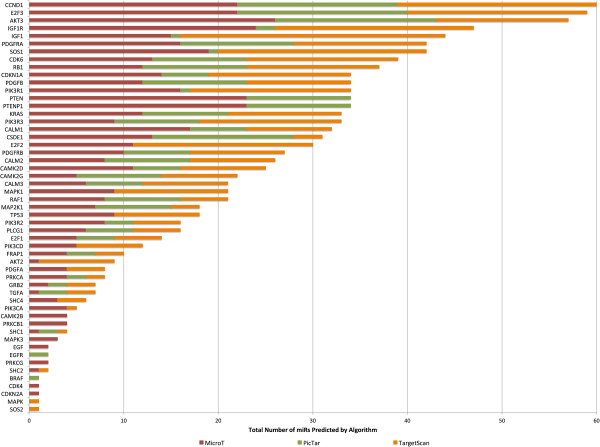
**Total number of miRs predicted by algorithm.** Illustration of which algorithms predicted the identified gene target by any of the 62 down-regulated miRs.

We employed the DIANA-mirPath [13] pathway analysis web-server to accomplish both target prediction and enrichment analysis. We used three gene target prediction algorithms in mirPath: TargetScan v5, PicTar 4-way, and DIANA MicroT v4 to analyze the datasets as separate jobs (Figure 2). After the gene targets were predicted, mirPath calculated the enrichment of genes in all biological pathways available in the KEGG database. After analysis, the user is presented with a visualization of any pathways that contained at least one gene. The pathways are ranked according to an enrichment significance score based on a Fisher’s combined probability (meta-analysis) test [13]. DIANA-mirPath also provides a union of pathways feature. Using this technique we were able to identify all significantly targeted pathways by the selected miRs. As above, the Fisher’s meta-analysis method was used to calculate *p*-values to illustrate the probability that the examined pathway is significantly enriched with gene targets of at least one selected miR [13]. For our list of 64 miRs, 18 enrichment pathways are highly significant (*p* < 1E-16). The glioma pathway is ranked as the 7^th^ most significant (*p* < 1E-16), and 11 of the 18 highest ranked pathways are cancer-related, such as endometrial cancer, colorectal cancer, prostate cancer, and bladder cancer. In order to examine the specificity of this approach we conducted the identical union of pathways analysis with a set of 64 randomly selected miRs. For this list of randomly selected miRs, the glioma pathway is not significant (*p* = 0.08). A total of 53 unique genes were identified as potential targets by the three prediction algorithms for glioma pathways (Table 3).

**Table 3:** Frequency that a gene is predicted by a miR by number of algorithms

	**Number of**				**Number of**		
	**Algorithms**				**Algorithms**		
Gene	3	2	1	Gene	3	2	1
CCND1	16	4	4	IGF1R	0	22	3
E2F3	12	11	1	SOS1	0	16	10
AKT3	11	9	6	PIK3R1	0	16	2
PDGFRA	11	4	1	IGF1	0	15	14
PDGFB	11	0	1	E2F2	0	11	8
RB1	10	2	3	PTEN	0	10	14
KRAS	9	3	0	PTENP1	0	10	14
PDGFRB	7	3	0	MAPK1	0	8	5
CDK6	6	8	5	TP53	0	8	2
CALM1	6	1	12	PIK3CD	0	5	2
CDKN1A	5	8	3	TGFA	0	3	1
PIK3R3	5	6	6	SHC4	0	3	0
CALM2	5	4	3	PDGFA	0	2	4
CAMK2D	5	4	2	PIK3CA	0	1	3
RAF1	5	3	0	SHC2	0	1	0
CALM3	5	2	2	AKT2	0	0	9
CAMK2G	5	1	5	CAMK2B	0	0	4
E2F1	4	1	0	PRKCB1	0	0	4
CSDE1	3	4	14	MAPK3	0	0	3
MAP2K1	3	4	1	EGF	0	0	2
PLCG1	3	3	1	EGFR	0	0	2
PIK3R2	3	2	3	PRKCG	0	0	2
FRAP1	3	0	1	BRAF	0	0	1
GRB2	2	0	1	CDK4	0	0	1
PRKCA	1	1	3	CDKN2A	0	0	1
SHC1	1	0	1	MAPK	0	0	1
				SOS2	0	0	1

**Figure 3:**
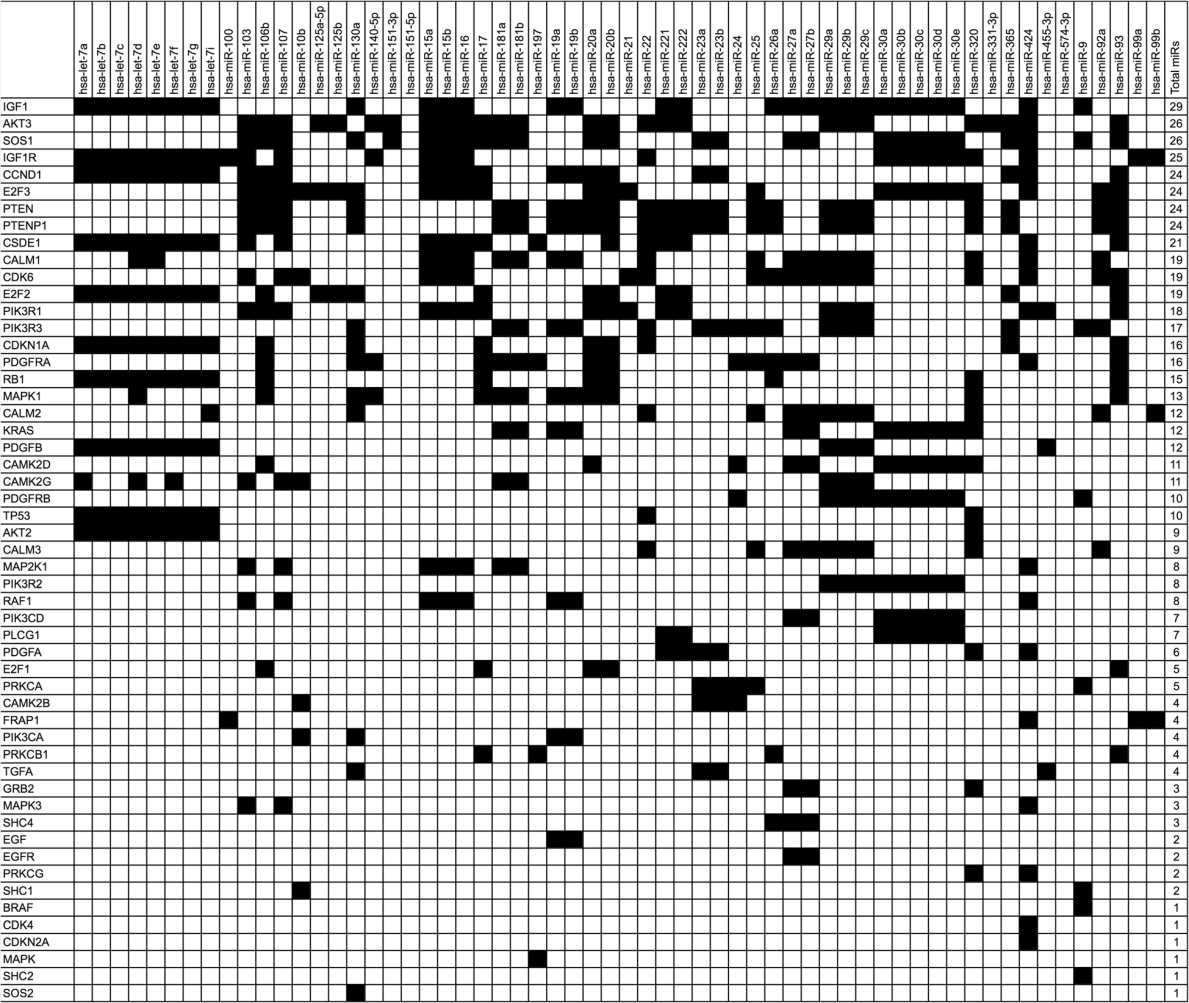
**Genes predicted by miR.** Illustration of the occurrences that a miR predicts one of the glioma pathway genes by any of the 3 target prediction algorithms.

**Figure 4:**
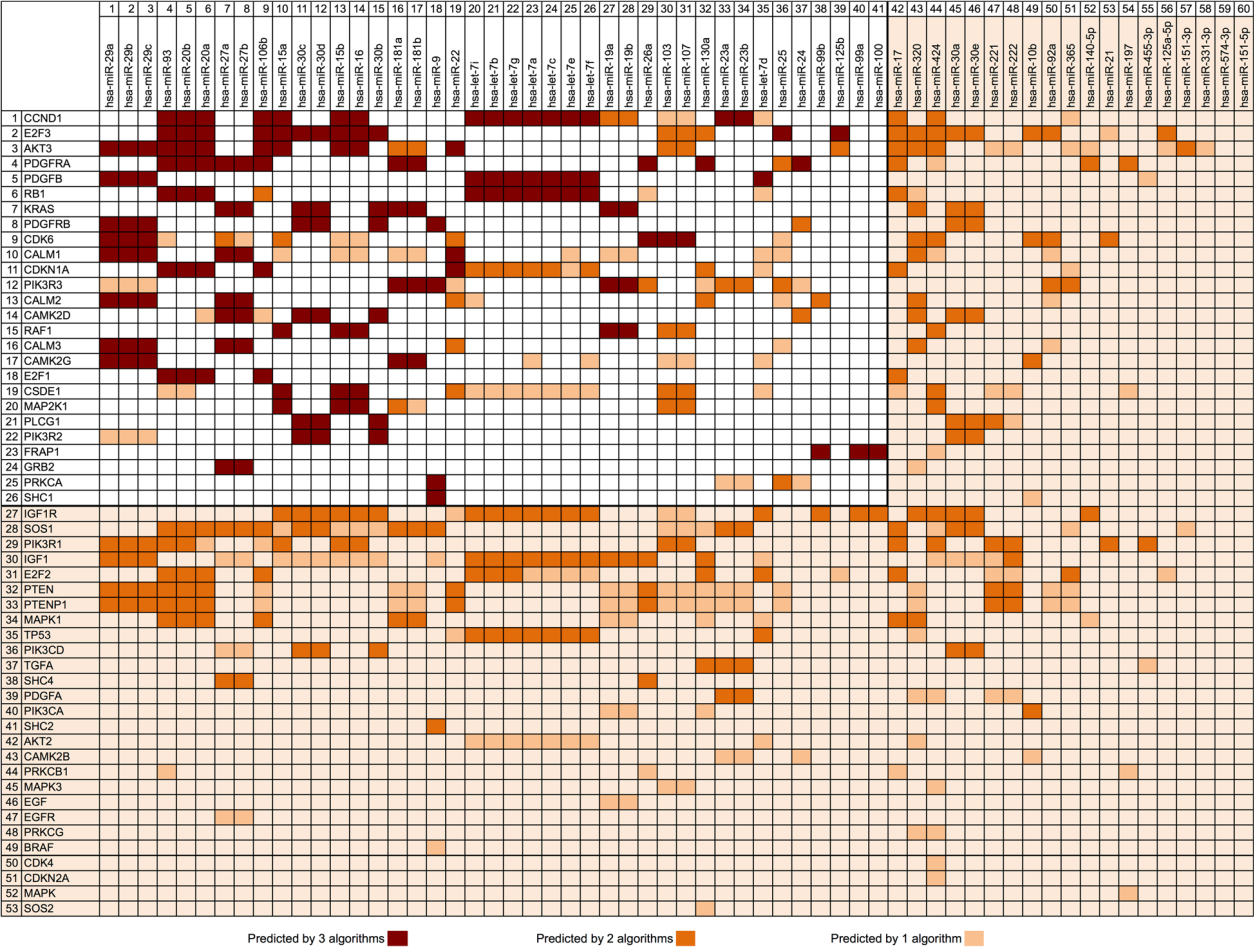
**Concurrences between glioma pathway gene target prediction algorithms.** Illustration of the 53 genes and 60 miRs identified. Area with white background illustrates the set of 26 genes and 41 miRs for which a gene is targeted unanimously by all 3 algorithms by 1 or more miR.

Despite the high rank of the glioma pathway reported by mirPath, we sought a more detailed view of the miR-gene interactions. We postulated that some genes might be preferentially targeted by multiple miRs in our dataset. Other studies employing miR pathway analysis favor comparing the results of multiple prediction algorithms to find consensus interactions [12]. Taking a similar approach, we recorded every potential miR-gene interaction among the glioma pathway for all three of the prediction algorithms (Figure 3). We summarized the findings with prediction consensus counts (from consensus of 0 algorithms to consensus of 3 algorithms) to identify the number of algorithms that predicted each miR-gene interaction. We preferentially focused our attention on interactions unanimously predicted by all three algorithms (score = 3). We then summed the number of unanimous interactions for each gene to assess the enrichment of single genes (Figure 4). This count provided an empirical indication that some genes are potentially targeted by many of the top miRs identified in our analysis. All 3 algorithms predict a glioma pathway gene target by 41 of the 62 down-regulated miRs in our study (Table 4).

**Table 4:** Frequency that a miR predicts a gene by number of algorithms

	**Number of**				**Number of**		
	**Algorithms**				**Algorithms**		
miR	3	2	1	miR	3	2	1
hsa-miR-29a	8	4	2	hsa-miR-107	1	6	7
hsa-miR-29b	8	4	2	hsa-miR-130a	1	6	7
hsa-miR-29c	8	4	2	hsa-miR-23a	1	4	4
hsa-miR-93	7	6	3	hsa-miR-23b	1	4	4
hsa-miR-20b	7	6	1	hsa-let-7d	1	3	9
hsa-miR-20a	7	5	2	hsa-miR-25	1	3	6
hsa-miR-27a	7	3	3	hsa-miR-24	1	2	3
hsa-miR-27b	7	2	4	hsa-miR-99b	1	2	0
hsa-miR-106b	6	4	4	hsa-miR-125b	1	1	1
hsa-miR-15a	6	3	3	hsa-miR-99a	1	1	0
hsa-miR-30c	6	3	1	hsa-miR-100	1	1	0
hsa-miR-30d	6	3	1	hsa-miR-17	0	11	2
hsa-miR-15b	6	2	4	hsa-miR-320	0	10	8
hsa-miR-16	6	2	4	hsa-miR-424	0	9	10
hsa-miR-30b	6	2	2	hsa-miR-30a	0	9	1
hsa-miR-181a	4	4	3	hsa-miR-30e	0	9	1
hsa-miR-181b	4	3	4	hsa-miR-221	0	4	5
hsa-miR-9	4	2	2	hsa-miR-222	0	4	5
hsa-miR-22	3	6	3	hsa-miR-10b	0	4	2
hsa-let-7i	3	5	3	hsa-miR-92a	0	3	5
hsa-let-7b	3	5	2	hsa-miR-365	0	2	6
hsa-let-7 g	3	5	2	hsa-miR-140-5p	0	2	2
hsa-let-7a	3	4	4	hsa-miR-21	0	2	1
hsa-let-7c	3	4	3	hsa-miR-197	0	1	3
hsa-let-7e	3	3	5	hsa-miR-455-3p	0	1	2
hsa-let-7f	3	4	4	hsa-miR-125a-5p	0	1	2
hsa-miR-19a	3	2	6	hsa-miR-151-3p	0	1	1
hsa-miR-19b	3	2	6	hsa-miR-331-3p	0	0	1
hsa-miR-26a	2	5	2	hsa-miR-574-3p	0	0	0
hsa-miR-103	1	6	7	hsa-miR-151-5p	0	0	0

## Discussion

Increased miR expression results in decreased messenger RNA (mRNA) expression, which in turn leads to decreased protein expression. Conversely, decreased miR expression could result in increased target mRNA expression, which in turn could lead to increased target protein expression. In the current study, we report the identification of a set of 62 miRs that exhibit statistically significant negative (down-regulated) differential expression in the migratory (edge) cell population relative to the corresponding expression in the matched migration-restricted (core) cell population. Bioinformatics analysis of potential targets of these down-regulated miRs produced a set of genes linked to regulation of apoptosis. Genes targeted by the down-regulated miR set have potential for increased expression in the invasive cell population and therefore represent potential therapeutic targets to limit glioma progression.

To begin our investigation of our miRs, we conducted an extensive literature review of verified gene targets relevant to cancer. The *let-7* family of miRs is well represented in our results. *Let-7* was one of the first two miRs identified and was shown to be a critical regulator of developmental timing [14]. The sequence of *let-7* was later discovered in the fruit fly and human genomes via BLAST search and became the first recognized miR in humans [15]. Interestingly, humans have 10 mature *let-7* isoforms that are produced from 13 distinct precursor sequences located at different locations in the genome [16]. Eight *let-7* family members were present in the set of 62 significant down-regulated miRs in the migratory cell invasive population.

*Let-7* members are widely considered critical tumor suppressors that, when lost, can alter cell growth and cancer progression [17]. In glioblastoma, transfection of *let-7 g* into U251 and U87 glioblastoma cells reduced the expression of Ras family proteins modulating proliferation and invasiveness [18]. Notably, increased expression of *let-7* inhibited *in vitro* proliferation and reduced tumor size in murine xenografts. Other studies have validated Ras as a target for let-7 family members [19]. In addition to let-7, our analysis also indicated that Ras proteins are potential targets for other miRs in our study: *miR-16, mir-27b, miR-30c* and *miR-15b* (Figure 3). It is well appreciated that Ras is an integral signaling constituent of many growth factor receptor pathways and that alterations in several growth factor receptor pathways, including EGFR and PDGFR, are a dominant characteristic of glioblastoma. As Ras signaling has been demonstrated to modulate glioblastoma cell proliferation [20], the loss of these four miRs, along with let-7, are likely to lead to altered Ras expression and activity. Target analysis also revealed that PDGFB is potentially targeted by many miRs in the study that together with PDGFR amplification provides a mechanism to potentiate tumor growth [21].

Two of the miRs in our study have been directly tied to neurological malignancies. *Let-7e* has been shown to inhibit neuroblastoma proliferation by targeting the MYC oncogene [22]. Meanwhile, *miR-181b* has been strongly implicated as a tumor suppressor in glioblastoma. Substantial down-regulation of *miR-181a* and *miR-181b* has been observed in both human glioma samples and in established glioma cell lines [23]. Expression of *miR-181* was abundant in normal brain tissue, but dropped substantially with increasing WHO grades [23]. Notably, transfection of *miR-181b* into glioblastoma cells significantly inhibited cell invasion in an *in vitro* matrigel invasion assay and increased apoptosis in the transfected cells [23].

Several miRs in our list appear to directly target apoptosis related genes. The anti-apoptotic protein BCL2 is a validated target of *miR-181b*[24] and *miR-16*[25]. Furthermore, BCL-XL is degraded by *let-7c*[20]. Elevated levels of these proteins desensitize cells to apoptosis, and thus it is likely that loss of these relevant miRs in migratory edge cells may cause increased expression of genes and lead to inhibition of apoptosis.

From the pathway enrichment analysis, we find that cyclin D1 (CCND1) stands out as a prominent target of a number of the miRs identified in this study (Figures 2, 3 and 4). Overexpression of the cyclin D1 protein is associated with tumorigenesis and is associated with poor outcome in a variety of cancers [26]. Cyclin D1 is a critical gene involved in the cell cycle control pathway. It is a regulatory subunit for the CDK4 and CDK6 proteins, and these kinases form active complexes that are required for a cell to progress from G1 to S phase. Cyclin D1 also binds to the retinoblastoma protein (RB1). RB1 is itself highly targeted by many of the miRs in our analysis (Figures 2, 3 and 4). The RB1 protein, on the other hand, opposes G1/S checkpoint transitions. It is thus interesting that both of these proteins are predicted targets by the same miRs in the identified set. This suggests a tightly controlled feedback loop that precisely regulates the balance between either cell cycle progression, or arrest, at G1. Perturbations of this balance lead to alterations in cell proliferation.

Mutations or aberrant expression of proteins in the cell cycle pathway have been associated with many cancers. In glioblastoma cell lines, it has been reported that cyclin D1 overexpression promotes invasiveness *in vitro*[27]. Furthermore, silencing cyclin D1 expression with siRNA inhibited invasion and apoptosis. *In vivo*, abnormalities in the cell cycle pathway are well recognized. In primary GB, the function of the tumor suppressor proteins p16-INK4A and p14-ARF is affected, whereas in secondary GB it is observed that CDK4 is amplified or RB1 is lost [27]. We speculate that loss of miRs targeting these proteins may lead to an overexpression of cyclin D1, which can deregulate the cell cycle in concordance with these other pathway abnormalities observed *in vivo*. Notably, our analysis identified a number of miRs that have been previously validated as targeting cyclin D1 including let7a-7f [28] as well as mir15 and miR16 [29]. If many of the other target predictions are valid, then we postulate these miRs may form an integral network involved in regulation of the cell cycle. Because cyclin D1 and RB1 are co-predicted by many of the same set of miRs, they may be appropriate targets for validation (Figures 2, 3 and 4).

Also among the miRNAs that was significantly down-regulated in the invasive cell population was miR-23b. A previous report demonstrated that miR-23b targets a set of genes associated with tumor invasion and metastasis [30] implicating its loss in facilitating tumor progression. Similarly, a recent report has demonstrated that miR-23b directly targets PIK3R3 [31] substantiating its identification and predicted targeting of PIK3R3 in the current analysis.

## Conclusions

Much progress in the understanding of miRs has been made over the past decade and research is identifying important functions for miRs in cancers such as glioblastoma. It is recognized that alterations in expression of miRNAs can significantly alter the proliferation and invasiveness of cells. Indeed, an increasing number of miRs have been validated as components of cancer-driving pathways. In this study we have applied a set of statistical and informatics tools and approaches, such as *t*-test, FDR, volcano plot, consensus miR target prediction and pathway analysis, to explore the role of miRs in glioblastoma. Approaches similar to the ones described here, that combine bioinformatics analysis of experimentally generated data with *in silico* miR target prediction and pathway enrichment analysis can be applied to other diseases to provide biomedical researchers and clinicians with increased opportunities for therapeutic interventions.

## Methods

### Data collection

RNA extraction and miR microarray profiling from matched sets of migratory (edge) and migration-restricted (core) cell populations of seven different glioblastoma cell lines has been described in detail [32]. The data were normalized to eliminate or reduce the potential for fluorescent intensity level bias. Control data was identified and removed before final analysis. The resulting cleaned dataset used in this study comprised 805 human miRs.

### Data analysis

A two-tailed *t*-test statistic (α = 0.05) and corresponding *p*-value was calculated for all miRs. SAS 9.2 was used for all statistical analysis. Statistical output for each analyzed miR included tests for normalcy to ensure appropriateness of analytical techniques. False discovery rate (FDR) correction was calculated using a Benjamini-Hochberg correction. A fold change method was used to identify differentially expressed miRs. This method evaluates the log ratio between two conditions; in this case the Log_2_ normalized expression levels of edge cell populations (migration cells) compared to core cell populations (migration-restricted cells). A twofold difference was used to identify meaningful differentially expressed miRs. In the transformed log scale, a twofold change corresponds to a 1.0 gene signal expression change. As this is not a true statistical test, no confidence levels can be attributed to the differentially expressed or non-differentially expressed miRs.

After calculation of *p*-values, FDR corrections, and log values, the data were further analyzed to identify miRs that exhibited both a significant corrected *p*-value and a greater than twofold change in expression level in the migratory edge cells relative to the migration restricted core cells. A total of 64 miRs satisfy the criteria of significant corrected *p*-value and ≥ twofold change in expression level. This subset of the data (64 miRs) represents the specific miRs identified for further study. Descriptive statistics were calculated for each of these 64 miR core and edge cell sample expression levels. Each miR was then assessed for dysregulation as either up-regulated (positive expression level) or down-regulated (negative expression level).

A volcano scatter plot method was used to graphically illustrate the relationship between significance levels for differentially expressed miRs. Negative Log_10_*p*-values for each of the 805 miRs were plotted on the y-axis and Log_2_ normalized fold change expression levels on the x-axis. The threshold Benjamini-Hochberg corrected -Log_10_*p*-value (1.687) was superimposed on the volcano plot for reference (horizontal dashed line) to identify miRs with significant differential expression (α = 0.05). In the resulting plot, miRs with a -Log_10_*p*-value of greater than the threshold corrected -Log_10_*p*-value (1.687), and a fold change of greater than twofold, comprise the 64 miRs identified for further study.

### Target prediction and pathway enrichment analysis

We employed the DIANA-mirPath website [13] for target prediction and pathway enrichment analysis for the 62 down-regulated miRs identified in the study. As the focus of the study is in migratory glioblastoma cells, we concentrated the majority of our effort on the down-regulated miRs (62) and included the up-regulated miRs (2) only as overall statistical measures in the analysis. We used three gene target prediction algorithms in mirPath: TargetScan v5, PicTar 4-way, and DIANA MicroT v4 to analyze the datasets as separate jobs. The mirPath tool includes a pathways union feature (*A Posteriori*), which we used to identify all significantly targeted pathways by the selected miR. The mirPath server performs the enrichment analysis and calculates the significance levels (*p*-values) for each selected miR. The tool then calculates a merged p-value for each pathway based on a Fisher’s combined probability test statistic (*X*^2^). The results from this meta-analysis method depict the probability that the examined pathway is significantly enriched with gene targets of at least one selected miR. We conducted this analysis for both our identified list of 64 miRs, as well as for a list of 64 randomly selected miRs in order to assess the specificity of our results.

### Availability of supporting data

Supporting data will be made available upon request.

## Abbreviations

DIANA: DNA intelligent analysis; FDR: False discovery rate; GB: Glioblastoma; Hsa: *Homo sapiens*; KEGG: Kyoto encyclopedia of genes and genomes; miR: microRNA; SD: Standard deviation.

## Competing interests

The authors declare that they have no competing interests.

## Authors’ contributions

BSB is the lead writer and conducted all data processing and analysis, JCL provided the experimental biological data and helped write the manuscript, CJM conducted pathway enrichment analysis, VD oversaw the study and provided overall guidance. All authors read and approved the final version of the manuscript.
